# Carcinome Hépatocellulaire (CHC)

**DOI:** 10.48327/mtsi.v4i4.2024.614

**Published:** 2024-12-18

**Authors:** Stanislas POL

**Affiliations:** AP-HP. Centre Université Paris Centre, Groupe hospitalier Cochin Port Royal, Département médical universitaire de Cancérologie et spécialités médico-chirurgicales, Service des maladies du foie, Paris, France; Université Paris Cité, F-75006, Paris, France

**Keywords:** Cirrhose, Carcinome hépatocellulaire, Virus de l'hépatite B, Virus de l'hépatite C/MASH, Cirrhosis, Hepatocellular Carcinoma, Hepatitis B Virus, Hepatitis C Virus/MASHIntroduction

## Abstract

Les cancers primitifs du foie sont des tumeurs développées à partir des différentes cellules hépatiques. Le carcinome hépatocellulaire (CHC), développé à partir des hépatocytes, représente environ 75 à 85 % des cancers primitifs du foie.

Le CHC représente la 6^e^ cause de cancer dans le monde et la 3^e^ cause de décès par cancer. Son incidence est faible en Europe du Nord, mais fréquente en Afrique subsaharienne ou en Extrême-Orient où non seulement les virus hépatotropes mais aussi les expositions aux mycotoxines sont d'incidence élevée. Il complique une cirrhose dans plus de 90 % des cas, avec une prédominance masculine.

La prévalence du CHC est en hausse en raison d'une amélioration des techniques et des critères diagnostiques, mais aussi de la persistance des infections par les virus de l'hépatite B (VHB) chez les adultes ou C (VHC). Une augmentation à l’échelle mondiale de l'incidence des stéatopathies en font la première cause d'hépatopathie dans le monde liées à l'abus d'alcool et/ou métaboliques (MASH pour metabolic dysfunction associated steato-hepatitis) incluant le diabète de type 2.

Infections virales chroniques hépatotropes, cirrhose et carcinogènes chimiques s'intriquent pour favoriser une incidence annuelle de 2 à 5 % de carcinomes hépatocellulaires survenant sur cirrhose. Ceci justifie la surveillance semestrielle des cirrhoses connues, en l'absence de laquelle les diagnostics tardifs limitent les possibilités thérapeutiques.

Les progrès ont été majeurs pour les traitements curatifs (transplantation hépatique, chirurgie, radio-destruction) ou palliatifs (chimio- ou radio-embolisation, chimiothérapie par sorafenib ou immunothérapie) qui dépendent de la précocité du diagnostic du CHC (taille, nombre de lésions hépatiques ou extra-hépatiques) et de la sévérité de la maladie hépatique sous-jacente et des comorbidités associées.

## Abstract (en)

Primary liver cancers are tumors that develop from different liver cells. Hepatocellular carcinoma (HCC), which develops from hepatocytes, accounts for approximately 75-85% of primary liver cancers.

HCC is the 6^th^ leading cause of cancer worldwide and the 3^rd^ leading cause of cancer-related death. Its incidence is low in northern Europe, but high in sub-Saharan Africa and the Far East, where both hepatotropic viruses and exposure to mycotoxins are. It complicates cirrhosis in over 90% of cases and is predominantly male.

The prevalence of HCC is increasing due to improved diagnostic techniques and criteria, but also to the persistence of hepatitis B virus (HBV) and hepatitis C virus (HCV) infections in adults. A worldwide increase in the incidence of steatopathy makes it the leading cause of liver disease worldwide, associated with alcohol abuse and/or steatohepatitis associated with metabolic dysfunction (MASH), including type 2 diabetes.

Chronic hepatotropic viral infections, cirrhosis and chemical carcinogens combine to produce an annual incidence of 2-5% of hepatocellular carcinoma arising from cirrhosis. This justifies biannual surveillance of known cirrhosis, without which late diagnosis limits therapeutic options.

Major advances have been made in curative treatment (liver transplantation, surgery, radiodestruction) and palliative treatment (chemo- or radioembolization, sorafenib chemotherapy or immunotherapy), depending on how early HCC is diagnosed (size, number of hepatic or extrahepatic lesions) and the severity of underlying liver disease and associated comorbidities.

## Introduction

Les cancers primitifs du foie sont des tumeurs développées à partir des différentes cellules hépatiques. Le carcinome hépatocellulaire, qui se développe à partir des hépatocytes, représente environ 75 à 85 % des cancers primitifs du foie [43, 57, 66]. Le cholangio-carcinome intra-hépatique ou extra-hépatique a une incidence croissante et représente environ 15 % des cancers; il est développé à partir des cellules biliaires. Il existe des formes mixtes d'hépato-cholangiocarcinome. L'hépatoblastome est une tumeur spécifique de l'enfant. L'angiosarcome ou l'hémangio-endothéliome épithélioïde sont rares et se développent à partir de la cellule endothéliale.

Le carcinome hépatocellulaire (CHC) représente en fréquence la 6^e^ cause de cancer dans le monde et la 3^e^ cause de décès par cancer [66]. Son incidence est faible en Europe du Nord, mais fréquente en Afrique subsaharienne ou en Extrême-Orient où non seulement les virus hépatotropes mais aussi les expositions aux mycotoxines sont d'incidence élevée. Il complique une cirrhose dans plus de 90 % des cas, avec une nette prédominance masculine. La prévalence du CHC est en hausse en raison d'une amélioration des techniques et des critères diagnostiques, d'une augmentation de son incidence dans le monde [57], comme conséquence des stéatopathies liées à l'abus d'alcool et/ou métaboliques (MASH pour *metabolic dysfunction associated steato-hepatitis)* incluant le diabète de type 2 [54, 64], de la stabilité des infections par le virus de l'hépatite B (VHB) qui augmente cependant chez les adultes malgré la vaccination et des infections par le virus C (VHC). L'incidence du CHC est supposée augmenter au cours des 20 prochaines années, surtout du fait de la MASH, notamment chez les femmes : les MASH sont devenues la première cause d'hépatopathie dans le monde [18, 54]. Les infections par les VHB ou VHC qui sont, parmi les rares virus humains avec les papillomavirus (HPV), le virus lymphocyto-tropique humain (HTLV) et certains virus de la famille herpes, liés à l'oncogenèse chez l'humain. Les mécanismes de l'hépatocarcinogénèse restent imprécis. Un certain nombre de facteurs carcinogènes ont été identifiés, pour lesquels il est difficile de distinguer les facteurs initiateurs des facteurs promoteurs. Les infections virales chroniques hépatotropes, la cirrhose elle-même et les carcinogènes chimiques s'intriquent pour favoriser l'incidence annuelle de 2 à 5 % de carcinomes hépatocellulaires survenant sur cirrhose.

## Épidémiologie

Le cancer du foie est le sixième cancer le plus fréquent, le cinquième chez l'homme et le septième chez la femme dans le monde, représentant environ 7 % du nombre total de diagnostics de cancer (environ 850 000 nouveaux cas chaque année) et cette incidence continue de croître. Il est la troisième cause de décès par cancer (environ 800 000 par an) [54, 66]. L'incidence annuelle du cancer du foie est très proche du nombre de décès par an qu'elle génère, ce qui souligne l'agressivité de cette maladie [66]. Le carcinome hépatocellulaire (CHC) représente 75 à 85 % de tous les cancers primitifs du foie. L'incidence de CHC est plus élevée chez l'homme, avec un ratio hommes-femmes de 3 [75]. Dans le monde, la mortalité liée au CHC est en hausse, malgré le caractère évitable de ses facteurs de risque. L'incidence et la mortalité du CHC devraient augmenter dans les pays africains, contraints par des ressources limitées pour lutter contre les niveaux endémiques d'infection virale et les facteurs de risque environnementaux synergiques [51]. La nature changeante de l’étiologie du CHC est particulièrement illustrée ici par les facteurs de risque traditionnels comme l'hépatite virale coexistant avec une prévalence élevée du virus de l'immunodéficience humaine (VIH) et une urbanisation croissante qui ont favorisé une forte augmentation de facteurs de risque supplémentaires comme la co-infection, le diabète sucré de type 2 et l'obésité [18, 57]. Bien qu'il existe certaines différences d’étiologie entre l’Afrique du Nord et l’Afrique subsaharienne, les facteurs de risque comme l'hépatite virale chronique B et C, l'exposition aux aflatoxines et la surcharge en fer prédominent. Les génotypes agressifs de l'hépatite B, associés aux co-infections par le virus de l'hépatite B/virus de l'hépatite C/VIH et à l'exposition aux aflatoxines, favorisent un phénotype moléculaire plus agressif. Parallèlement à une meilleure compréhension de l’étiologie moléculaire du CHC, les initiatives politiques et de planification visant à faire face au fardeau du CHC doivent être ancrées dans la réalité des ressources limitées disponibles. La création et la coordination de registres du cancer à travers l’Afrique sont importantes pour améliorer la qualité des données nécessaires pour galvaniser l'action. Les mesures préventives, notamment les programmes de vaccination contre l'hépatite B [68], les mesures de prévention de la transmission de la mère à l'enfant et de l'enfant à l'enfant, la fourniture de traitements antirétroviraux et antiviraux universellement accessibles [11, 13] et la réduction de l'exposition alimentaire aux aflatoxines peuvent contribuer de manière significative à réduire l'incidence du CHC [52]. Enfin, le développement de biomarqueurs et de nouvelles interventions thérapeutiques nécessitera une meilleure compréhension des caractéristiques génétiques et épigénétiques uniques du CHC sur le continent africain.

## Facteurs de risque

Les facteurs de risque de CHC sont bien connus. Le CHC survient le plus souvent chez des patients ayant une fibrose hépatique avancée ou une cirrhose, facteur de risque principal; elle est due à une maladie chronique du foie, causée principalement par la consommation chronique excessive d'alcool, la stéatohépatite non alcoolique MASH, le virus de l'hépatite B (VHB) ou le virus de l'hépatite C (VHC) [8]. Les taux les plus élevés d'incidence de CHC se trouvent en Asie (Chine, Mongolie) et en Afrique subsaharienne. Dans l'ensemble, 54 % des cas pourraient être attribués à l'infection par le VHB, 31 % à l'infection par le VHC et 15 % à d'autres causes, mais l’épidémiologie du CHC évolue : alcool et MASH restent les causes les plus fréquentes dans les pays du Nord, et les infections virales chroniques B, C ou D dans les pays du Sud [53].

Le CHC est responsable de la majorité des décès des patients cirrhotiques [4]).

La cirrhose résulte de différents mécanismes d'atteinte hépatique qui entraînent une inflammation et une fibrogenèse [67]. Histologiquement, elle est caractérisée par une régénération nodulaire diffuse entourée de septa fibreux avec extinction du parenchyme qui provoque une distorsion de l'architecture vasculaire hépatique. Il en résulte une hypertension portale et une insuffisance hépatique [67].

La cirrhose est le principal facteur de risque pour le développement du CHC et environ 30 à 35 % des patients cirrhotiques développeront un CHC avec un risque annuel d'environ 1 à 8 %, en fonction de l’étiologie de la cirrhose [34]. Le CHC peut se développer à tous les stades de la cirrhose qu'elles qu'en soient leurs causes [24]. La classification de Child-Turcotte-Pugh est largement utilisée pour caractériser le degré d'atteinte hépatique et prédire le pronostic des patients atteints de cirrhose [67]. Les recommandations sont d'effectuer une échographie hépatique semestrielle pour le dépistage précoce du CHC car les traitements des plus petits CHC sont les plus efficaces [8, 24, 72]. Le syndrome métabolique [47, 72] et la consommation de tabac [37] sont des cofacteurs du risque de cancer du foie. Le sexe masculin, l’âge > 60 ans ou une maladie active [24] participent du risque de CHC. Certains facteurs de protection ont été décrits, tels que les médicaments (statines, antidiabétiques comme la metformine, aspirine) ou des facteurs alimentaires (café, vitamine E) [63].

La MASH et l'infection par le VHB sont les deux principales causes de développement d'un CHC sans cirrhose [64, 76]. Il est suggéré qu'environ 40 % des patients avec CHC sur MASH pourraient ne pas avoir de cirrhose [47].

## Physiopathologie

### La cirrhose, facteur de risque principal de CHC

L'histoire naturelle du CHC dans la cirrhose suit une chaîne d’événements, depuis le développement de nodules cirrhotiques précancéreux en nodules de dysplasie de haut grade, pouvant ensuite conduire à l'apparition d'un CHC à un stade précoce (stades 0 et A) puis progresser vers un CHC plus avancé (stades B et C) [43, 57]. Le CHC résulte de l'accumulation de mutations somatiques d'oncogènes. Dans les nodules de CHC, une accumulation de mutations dans les régions codantes d'environ 40 gènes fonctionnels somatiques peut être observée, révélant l'importante diversité du CHC [59]. Certains marqueurs discriminants entre la tumeur précancéreuse et le CHC ont été identifiés : activation de la voie WNT–ß-caténine, réexpression de gènes fœtaux, dérégulation de mécanismes de repliement des protéines, réponse au stress oxydatif et maintien de la télomérase [38, 49]. Les changements génétiques et épigénétiques sous-tendent la transformation oncogénique. Ils peuvent déréguler l'expression d'antigènes oncofœtaux qui deviennent des antigènes associés aux tumeurs (AAT) capables d'induire des réponses immunitaires [28, 55]. Deux d'entre eux sont l’α-foetoprotéine (AFP) et le glypican-3 (GPC3). Le CHC est un cancer associé à l'inflammation avec une association très élevée (90 %) à une hépatite chronique, quelle qu'en soit la cause, notamment une infection virale, une consommation excessive d'alcool ou une MASH, indiquant que le microenvironnement joue un rôle majeur dans la pathogenèse de la maladie [30]. Bien que la présence d'un infiltrat immunitaire soit associée à un meilleur pronostic [70], l'activation du facteur nucléaire kB (NF-kB), du facteur de croissance épidermique (EGF) et de l'interleukine-6 (IL-6) dans le parenchyme hépatique et non dans les cellules tumorales est liée à un mauvais pronostic [31].

Le cancer est une maladie de l’ADN. La transformation maligne d'une cellule est secondaire à des altérations génétiques et/ou épigénétiques de gènes contrôlant le cycle, la prolifération cellulaire et la survie des cellules. L'apparition de mutations « donnant l'avantage » favorise l’émergence de clones hépatocytaires dont la prolifération accrue favorise les nouvelles mutations.

On peut schématiquement classer les mécanismes de transformation hépatocytaire en deux groupes en fonction de leur émergence sur foie cirrhotique ou non cirrhotique. Le premier groupe, de loin le plus fréquent, survient dans un contexte nécrotico-inflammatoire chronique, de stress génotoxique et de régénération. La réponse normale d'une cellule aux altérations de son ADN est un arrêt du cycle. À l’échelon d'un organe, la conséquence de l'arrêt du cycle des hépatocytes est un défaut de régénération. Parfois, les barrières naturelles sont franchies, favorisant l’émergence de clones hépatocytaires plus ou moins dysplasiques. Les altérations génétiques observées à ce stade sont des pertes ou des gains de chromosomes entraînant des altérations de l'expression de p53, des mutations de bêta-caténine et d’AXIN responsables d'une activation de la voie Wnt/bêta-caténine, des inactivations de pRb1, IGF2R et de p16. L'apparition du CHC chez les patients cirrhotiques dépend de l'activité, de la durée et de l’étiologie de la maladie hépatique initiale et les nodules dysplasiques et macro-nodules de régénération doivent être considérés comme des lésions pré-cancéreuses [8, 75] :

1. l’émergence de CHC au sein de nodules cirrhotiques est classique (aspect de « nodule dans le nodule »);
2. 50 à 60 % des macro-nodules cirrhotiques ont une composante monoclonale si l'on étudie le profil de méthylation du chromosome X;
3. des aberrations chromosomiques et des pertes d'allèles sont trouvées dans la moitié des nodules cirrhotiques et dans les nodules dysplasiques.

### Les CHC sans cirrhose

Les rares cas de CHC sur foie sain sont spécifiques de certains mécanismes et de certains pathogènes : mutagenèse insertionnelle et VHB, mutation R249S de p53 et exposition à l'aflatoxine B1mutation kRAS et au chlorure de vinyle, mutation du facteur de transcription HNF1a et adénomes hépatocytaires, mutations germinales du gène APC et hépatoblastomes.

On peut également classer les mécanismes transformants selon la présence ou l'absence d'instabilité chromosomique. Les CHC secondaires au VHB sont souvent peu différenciés et liés à une instabilité chromosomique et à une mutation fréquente de p53. Les CHC non liés au VHB sont souvent bien différenciés, dénués d'instabilité chromosomique, et souvent associés à des mutations de la voie wnt/beta-caténine.

### Hépatocarcinogénèse virale

#### Preuve épidémiologique du lien VHB/CHC

L'association entre les infections par le virus de l'hépatite B (VHB) et le CHC est suggérée par :

1. l'augmentation d'incidence de ce cancer dans les zones d'endémie virale avec un risque relatif de 10 à 100 dans les zones d'endémie [53, 73];
2. les modèles animaux d'infections par les hepadnavirus (particulièrement le virus de la marmotte);
3. le lien entre charge virale initiale et CHC [32] et la diminution significative de l'incidence de la maladie virale B par les politiques de vaccination systématique des nouveau-nés et des adolescents [5]. La vaccination des nouveaunés de mères infectées puis des adolescents puis la vaccination universelle ont permis une réduction de 85 % du portage chronique chez les nouveau-nés de mères infectées et de 50 % de la prévalence du portage chronique de l'antigène HBs en Europe et aux États-Unis, de la morbidité et de la mortalité rapportées à l'infection. Parallèlement, ces dix dernières années, la fréquence du CHC dans les zones de haute endémie (Hong Kong, Singapour, Taiwan) ont chuté d'environ 50% grâce à ces politiques de vaccination [5]. Ceci est la première démonstration d'une prévention du cancer par un vaccin. Parallèlement, le lien causal entre niveau de multiplication virale et CHC a été clairement établi par l’étude taïwanaise « Reveal » [13] et par l'efficacité de la virosuppression par les analogues nucléosidiques par la réduction de l'incidence du CHC d'autre part [2, 65, 68, 73].

#### Mécanismes de l'hépato-carcinogénèse virale B

Les mécanismes de l'hépato-carcinogénèse virale B sont complexes [5]. Ils associent, du fait de l'intégration du génome viral (autorisée par une étape de réverse transcription dans la multiplication du VHB), des réarrangements chromosomiques, des mécanismes de cis-activation d'une part et de trans-activation d'autre part. La variabilité du VHB a aussi été mise en cause (génotype A, délétion dans le domaine pré S2 entre les nucléotides 38-55 du VHB [19].

Les mécanismes de cis-activation sont représentés par la mutagenèse insertionnelle (cis-activation d'un proto-oncogène cellulaire) décrite pour l'insertion dans le gène de la Cycline A, le gène du récepteur de l'acide rétinoïque et le gène codant pour la mévalonate kinase humaine. Des phénomènes de trans-activation sont probables : ainsi, la protéine X du VHB pourrait jouer un rôle de transactivation sur de nombreux promoteurs hétérologues ou homologues.

Bien que les mécanismes d'hépato-carcinoge-nèse virale B soient acquis, il est probable qu'ils interagissent avec d'autres facteurs étiologiques : l'alcool, l'aflatoxine B1, le virus de l'hépatite C ou l'hépatite chronique et la cirrhose elle-même, comme dans les autres hépatopathies en particulier l'hépatite C.

#### Hépato-carcinogénèse virale C

Certains CHC ont été observés chez des patients n'ayant pas d'antigène HBs (Ag HBs) détectable, suggérant l'intervention d'autres virus que le VHB, même si la mise en évidence de séquences intégrées du VHB dans un nombre non négligeable de cas souligne l'origine virale B possible de certains CHC survenant en l'absence d’Ag HBs. Différents arguments, principalement épidémiologiques, suggèrent que le virus de l'hépatite C (VHC) pourrait favoriser l’émergence de CHC par des mécanismes différents du VHB du fait de l'absence d'intégration dans le génome de l'hôte. La plus grande fréquence des anticorps dirigés contre le VHC chez les patients ayant un carcinome hépatocellulaire (30 à 70 %) que dans la population générale (1 %), en l'absence d’Ag HBs détectable, est un argument épidémiologique. Les mécanismes de l'hépato-carcinogenèse virale C restent inconnus et sont probablement multifactoriels : l'inflammation chronique associée à l'infection virale, la fibrose hépatique qui en est la conséquence et dont le stade final est la cirrhose, jouent un rôle majeur. L'importance de ces facteurs est illustrée par le développement dans environ 90 % des cas du CHC sur des lésions d'hépatite chronique et de cirrhose.

Cependant, un rôle direct du VHC est suggéré par la détection *in vivo* de séquences d’ARN VHC (brins positifs et négatifs) dans les cellules tumorales, ou par le développement d'un CHC associé à l'expression de la capside dans le foie de souris transgéniques. *In vitro,* l'expression stable de la protéine non-structurale NS3 transforme des cellules NIH 3T3, et l'expression de la capside VHC, associée à l'oncogène Ha-ras, pourrait transformer des fibroblastes embryonnaires de rat. Plusieurs protéines virales du VHC, notamment la protéine Core du virus, NS3 et NS5A ont été directement incriminées dans la transformation et le développement du CHC [69].

Le stress cellulaire associé à l'infection virale C chronique active des voies de signalisation cellulaire comme les voies MAPK qui facilitent la prolifération cellulaire et favorisent la transformation; il conduit à une accumulation intra- et extra-cellulaire d’éléments mutagènes pouvant induire des lésions de l’ADN de la cellule hôte.

#### Hépato-carcinogénèse virale D

Le virus de l'hépatite D (VHD) est un petit virus à ARN défectueux qui dépend du virus de l'hépatite B (VHB) pour l'assemblage et la transmission du virion. Il se réplique dans le noyau des hépatocytes et interagit avec plusieurs protéines cellulaires [12]. L'hépatite D chronique est une maladie grave et progressive, conduisant à une cirrhose dans 80 % des cas, responsable de la mortalité par décompensation hépatique ou carcinome hépatocellulaire [1, 26]. Savoir si le VHD est un virus oncogène reste débattu. Des études menées au cours de la dernière décennie ont montré que le VHD est associé à un risque significativement plus élevé de développer un CHC par rapport à la mono-infection par le VHB [1, 12, 26, 62]. Cependant, les mécanismes par lesquels le VHD favorise le cancer du foie restent incertains en dehors du fait que la prévalence de la cirrhose est plus élevée. Des données récentes montrent que le profil moléculaire du CHC associé à l'infection VHD est unique et distinct de celui du CHC associé à l'infection VHB, avec un enrichissement des gènes impliqués dans la réplication du cycle cellulaire et la réplication de l’ADN et dans les dommages et la réparation de l’ADN, ce qui indique que l'instabilité du génome est un mécanisme important de l'hépatocarcino-genèse au VHD [25]. Ces données suggèrent que le VHB et le VHD favorisent la carcinogenèse par des mécanismes moléculaires distincts malgré la dépendance obligatoire du VHD au VHB.

#### Autres carcinogènes

Des carcinogènes favorisent la maladie hépatique, c'est-à-dire la fibrogenèse et le risque de CHC (ou de cholangiocarcinome ou d'hépato-cholangiocarcinome).

L'alcool en est le principal [60] : la cirrhose alcoolique est associée à un risque de l'ordre de 10 % de développement de cancer primitif du foie. Le risque relatif chez les buveurs excessifs est de l'ordre de 1,2 à 4,2. Comme co-carcinogène, du fait de la dénutrition, de la fréquente exposition aux virus hépatotropes liée à l'alcoolisation chronique, la consommation excessive d'alcool expose à un risque accru de carcinome hépatocellulaire. La MASH par le biais de l'activité nécrotico-inflammation et de la fibrose en est un autre, particulièrement lorsqu'un diabète de type 2 est présent [64, 72].

Le facteur de transcription ChREBP apparait comme un oncogène hépatique. C'est un coordinateur métabolique central, et notamment un régulateur majeur dans le contrôle transcriptionnel du flux de glucose par la lipogenèse de novo dans le foie et le tissu adipeux blanc. La dérégulation des fonctions de ce modulateur clé de la communication inter-organes [56] est associée au développement de maladies métaboliques telles que le diabète de type 2 et la MASH [3]. Sa surexpression dans les modèles murins comme chez l'homme participe des mécanismes d'initiation et de développement du CHC et son invalidation réduit la croissance tumorale. Il pourrait être un nouveau biomarqueur moléculaire capable de prédire la résistance aux traitements systémiques utilisant des inhibiteurs de multikinases.

Des carcinogènes chimiques ont été identifiés comme favorisant la survenue de la tumeur primitive du foie. La mycotoxine aflatoxine B1 est la plus fréquemment décrite; les zones à haute exposition aux aflatoxines sont superposées à celles de haute endémie pour le VHB. Il existe une probable interaction entre les carcinogènes chimiques et les virus hépatotropes. Ainsi, chez les sujets Ag HBs positifs, exposés à l'aflatoxine, il a été montré que l'aflatoxine B1 pouvait provoquer l'apparition d'une mutation ponctuelle au niveau d'un gène suppresseur de tumeurs ou «anti-oncogène» : la P 53 [5, 74].

D'autres carcinogènes chimiques tels que les nitrosamines (dans les modèles animaux), le chlorure de vinyl (angiosarcomes) ou les agents permettant la prolifération des peroxysomes (dans les modèles animaux) peuvent favoriser l'apparition de tumeurs primitives du foie.

En résumé, toutes les étiologies des maladies hépatiques chroniques favorisent la fibrose extensive et la cirrhose, cause principale du CHC, et les infections par le VHB (et probablement par le VHC) sont directement impliquées dans la carcinogénèse hépatique. En ce qui concerne les hépatites auto-immunes qui peuvent également évoluer vers la cirrhose, il a été récemment montré que le risque de CHC dans cette population était faible [20].

Plusieurs mécanismes, qui ne sont pas exclusifs, sont probablement intriqués chez un même malade : la cirrhose, secondaire à une hépatite chronique active ou à une intoxication alcoolique chronique, est un facteur de risque majeur, associé dans plus de 90 % des cas à la tumeur. Le virus de l'hépatite B peut avoir un effet direct du fait de l'intégration avec réarrangement chromosomique ou mutagénèse insertionnelle, ou par la transactivation de protéines virales générées à partir d’ADN du virus de l'hépatite B en cours de multiplication ou intégré dans l’ADN cellulaire. Le VHD, satellite de l'infection par le VHB, augmente d'un facteur 2 à 3 le risque de cirrhose et par là-même le risque de CHC. Le VHC peut aussi directement intervenir par le biais de protéines virales transformantes.

La transformation maligne des hépatocytes survient en général dans un contexte de lyse et de régénération hépatique chronique et le plus souvent sur foie cirrhotique. La prolifération cellulaire, l'inflammation et le stress génotoxique facilitent l'accumulation d'anomalies génétiques et épigénétiques pouvant :

1. « allumer » des oncogènes ou « éteindre » des anti-oncogènes;
2. modifier l'expression de protéines régulatrices du cycle cellulaire;
3. réactiver la télomérase dans des hépatocytes sénescents.

## Diagnostic

### Diagnostic morphologique

Le CHC est au mieux diagnostiqué dans le suivi semestriel échographique d'un patient à risque car ayant une fibrose extensive ou une cirrhose précédemment diagnostiquée [24, 67, 72]. Ce dépistage échographique précoce du CHC, recommandé unanimement par les sociétés savantes, autorise d'espérer un traitement curatif du CHC dans plus de 70 % des cas. En l'absence d'hépatopathie connue, le diagnostic peut être fortuit ou effectué du fait d'une altération de l’état général qui révèle et la maladie hépatique fibrosante (dysmorphie, voire hypertension portale (HTP) et/ou ascite) et le cancer (nodule hypoéchogène dont la taille et le nombre sont précisés, de même que la perméabilité des axes vasculaires, notamment du tronc porte). Le pronostic est alors plus sombre et la curabilité de la tumeur incertaine (amélioration de 37 % de survie à 4 ans chez les patients dépistés par rapport aux non dépistés [77, 78].

Le diagnostic de CHC peut être posé par des méthodes non invasives (radiologie) et/ou invasives (biopsie).

Le diagnostic radiologique est acquis avec un degré de confiance élevé si la lésion survient chez un patient suivi pour une cirrhose. L’échographie va conduire à une imagerie dynamique de contraste (en utilisant une imagerie dynamique, échographie avec injection de produit de contraste, tomodensitométrie (TDM) ou imagerie par résonance magnétique (IRM)) : la lésion doit répondre aux critères radiologiques du CHC, c'est-à-dire une hypervascularité en phase artérielle *(wash-in)* et une diminution de signal par rapport au reste du parenchyme hépatique en phase tardive (lavage ou *wash-out)* [46]. Lorsque ces caractéristiques typiques sont présentes, une confirmation diagnostique par une biopsie n'est généralement pas nécessaire [8, 72], d'autant plus si l'alpha-foetoprotéine (AFP) est > 250 U/ml. Néanmoins, l’évolution dans la compréhension de la biologie moléculaire des tumeurs pourrait la rendre indispensable à l'avenir pour des classifications pronostiques et des thérapies ciblées [50]. La biopsie est indispensable chez les patients ne présentant aucun risque particulier de CHC, c'est-à-dire les patients sans cirrhose en foie tumoral et non tumoral.

**Figure 1:**
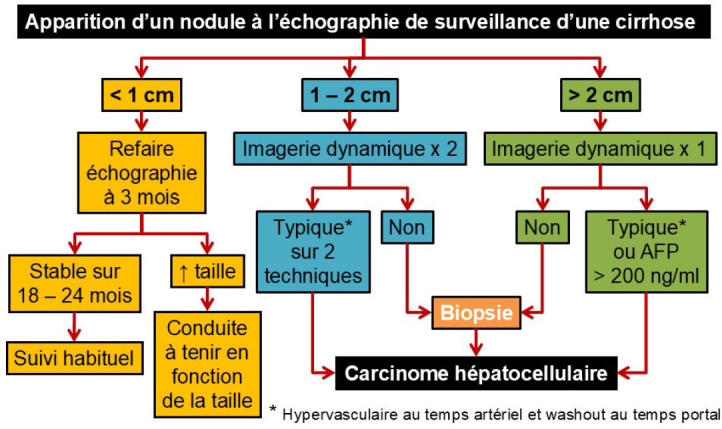
Démarche diagnostique du carcinome hépatocellulaire en cas d'apparition d'un nodule hépatique à l’échographie semestrielle de dépistage

L'algorithme recommandé pour l'investigation de lésions hépatiques chez les patients à risque est : pour les nodules > 1 cm, un suivi par échographie à 3 mois; pour les lésions > 1 cm, les signes radiologiques du CHC définissent le diagnostic; si la radiologie n'est pas typique dans au moins une des deux techniques d'imagerie (scanner et IRM), une biopsie du foie tumoral et non tumoral sous contrôle radiologique (majoritairement écho-guidée pour les lésions échogènes) est recommandée [24, 72] (Fig. 1).

Les difficultés diagnostiques du CHC et de sa prise en charge dans les pays à ressources limitées doivent être rappelées. C'est d'autant plus regrettable que, pour des raisons épidémiologiques (VHB notamment), la prévalence du CHC est au moins 10 fois plus fréquente que dans les pays du Nord [51]. Une évolution de l’épidémiologie du CHC est observée en Afrique. L'analyse de 863 patients ivoiriens ayant un CHC diagnostiqué entre 2007 et 2014 a montré des variations de présentation par rapport à la période 1970-1980 [22]. L’âge de présentation est retardé (49,4±14,1 ans) et le sex-ratio a diminué (M:F=2,6). Les patients séropositifs pour l'antigène de surface de l'hépatite B et anti-VHC représentaient 65 % et 25 % des cas tandis que la consommation d'alcool était rapportée dans 36 % des cas. Le taux d’AFP était supérieur à 400 ng/ml dans 36 % des cas et les tumeurs étaient déjà multinodulaires et/ou métastatiques au diagnostic chez 77 % et 26 % des patients. Des variations géographiques et anthropologiques ont été observées avec des excès de cas féminins affectant les régions (Lagunes) ou les groupes linguistiques (Kru). Les locuteurs du Nord Mandé étaient plus souvent identifiés comme nonB-nonC que les autres. L’évolution épidémiologie ivoirienne du carcinome hépatocellulaire ces dernières décennies est probablement due à la propagation du VHC (population de patients plus âgée et féminisée), ce qui risque de limiter les bénéfices attendus de la vaccination anti-hépatite B et justifierait des mesures appropriées pour prévenir de nouvelles transmissions du VHC.

Les retards au diagnostic et au traitement expliquent les différences considérables de pronostic du CHC entre les pays du Sud et les pays du Nord. L'absence de dépistage large des infections virales hépatiques expliquent dans ces situations la sousestimation des diagnostics d'infection virale chronique, d'hépatopathie et de cancer. Il y a un accès limité aux tests diagnostiques sérologiques et génomiques, peu de « *reflex testing* », c'est-à-dire qu'une sérologie positive - AgHBs, anti-VHD ou VHC - va conduire à une recherche des antiVHD et à la quantification de l’ADN VHB, de l’ARN VHD ou VHC. L’évaluation de la fibrose hépatique par des tests non invasifs de fibrose (Fibrotest, Fibroscan) est rarement disponible, même si des tests simples et peu coûteux, comme le FIB4, devraient permettre l’évaluation de la fibrose significative en population générale et donc identifier le risque de cirrhose et de CHC. Enfin, l'accès à une imagerie diagnostique simple et efficace comme l’échographie n'est pas toujours possible. Ceci explique que le diagnostic soit souvent fait à un stade avancé [22], dépassant toute possibilité thérapeutique curative. Les coûts des tests virologiques ou de biologie simple (alphafétoprotéine) et ceux d'une imagerie, de qualité variable, limitent significativement l'optimisation de la cascade de soins du dépistage au traitement.

### Stadification

**Figure 2:**
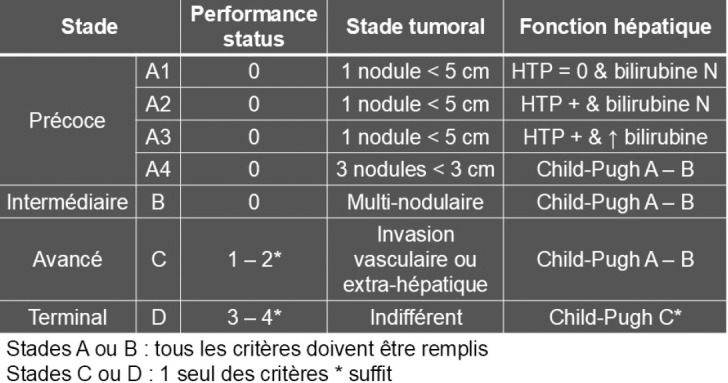
Système de stadification de Barcelone BCLC et stratégie thérapeutique. La classification comprend cinq étapes pour sélectionner les meilleurs candidats pour les meilleures thérapies actuellement disponibles. Les patients atteints de tumeurs précoces asymptomatiques (stades 0 – A) sont candidats à un traitement radical (résection, transplantation hépatique ou ablation locale). Les patients asymptomatiques avec CHC multinodulaire (stade B) requièrent un traitement par chimio-embolisation transartérielle (TACE), alors que les patients présentant une tumeur symptomatique avancée et invasive (stade C) sont des candidats pour recevoir une immunothérapie plus que le sorafenib. Pour les patients au stade terminal (stade D), seuls les soins palliatifs leur sont proposés.

Les principaux facteurs pronostiques de la survie chez les patients atteints de CHC sont la fonction hépatique, les caractéristiques de la tumeur (taille et nombre de nodules de CHC, invasion vasculaire), le taux sérique d’AFP et l’état général OMS [24, 72]. Le système de stadification le plus répandu et accepté en oncologie est la classification des tumeurs malignes (TNM). Le problème de ce système est qu'il ne prend pas en compte la réserve de fonction hépatique, ce qui est essentiel dans le CHC. L'association européenne pour l’étude du foie (EASL) et l'association américaine pour l’étude des maladies du foie (AASLD) [24, 72] approuvent la classification de Barcelone (BCLC) et recommandent l'utilisation de ce système de stadification pour évaluer le pronostic des patients et leur allouer le traitement adapté [39]. La classification BCLC divise les patients en cinq stades (0, A, B, C, D) (Fig. 2) en fonction de variables pronostiques préétablies liées :

1. à la tumeur : taille et nombre de nodules de CHC, invasion vasculaire ou ganglionnaire, extension extra-hépatique appréciées par l'imagerie (scanner thoracique, IRM, plus que TEP-scanner au FDG18 ou à la choline dont l'acuité diagnostique est >50 %);
2. au patient : état général, grade OMS et comorbidités;
3. à la sévérité de la maladie hépatique biologique (TP, albumine, bilirubine pour la mesure de la fonction hépatique, créatininémie et stade de Child-Pugh ou Meld si une transplantation hépatique est envisagée), avec ou sans hypertension portale (HTP) : plaquettes, fibroscan voire endoscopie oeso-gastrique à la recherche de varices oesophagiennes ou cardio-tubéro-sitaires selon les critères de Baveno.

La synthèse de l’évaluation de la sévérité du CHC (hépatique seulement ou plus étendu en hépatique et extra-hépatique), de l’état général du patient et de la sévérité de son hépatopathie vont conduire à la décision de traitement en réunion de concertation pluridisciplinaire (RCP).

## Traitements du CHC

**Figure 3:**
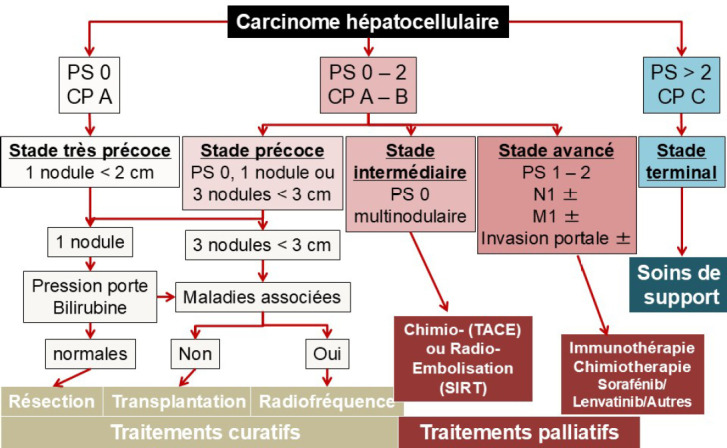
Choix thérapeutiques selon le stade BCLC du carcinome hépatocellulaire

Les traitements sont en progrès constants et décidés de façon collégiale en RCP. Selon la sévérité des patients, des traitements curatifs ou palliatifs peuvent être disponibles (Fig. 3).

### Traitements curatifs

Ce sont la radio-destruction tumorale transcutanée, la chirurgie de résection ou la transplantation hépatique. Pour la maladie à un stade précoce (BCLC 0 et A), la résection chirurgicale est l'option de première intention en cas de nodule solitaire et confère 70 % de survie à 5 ans [6, 24, 72]. Complications (mortalité post-opératoire > 5 %) et récurrence ont été réduites avec une sélection restrictive des candidats : nodule unique, absence d'hypertension portale et fonction hépatique préservée [24, 54, 72]. Une récidive de CHC et/ou l'apparition de métastases surviennent dans 70 % des cas 5 ans après la résection, et aucun traitement adjuvant ne peut pour l'instant l'empêcher [33].

Pour les patients BCLC A avec les critères de Milan (nodule unique de taille ≤ 5 cm ou jusqu’à 3 nodules d'une taille inférieure ou égale à 3 cm, sans invasion vasculaire) avec un score AFP (taille, nombre de nodules, valeur d’AFP) >2 [21] et récusés pour la résection chirurgicale (hépatopathie trop sévère), la transplantation hépatique (TH) est le meilleur traitement de première intention [42] avec un taux de survie respectif à 5 et 10 ans de 70 % et supérieur à 50 %, et avec un taux de récidive d'environ 10 % [17, 24, 42, 45, 72]. En réalité, 10 % des CHC sont discutés pour la transplantation et moins de 5 % des CHC sont transplantés [45]. Le CHC est l'indication du tiers des 1 300 TH effectuées en France chaque année [45]. On réalise le plus souvent la TH après une première récidive du CHC traitée par radio-ablation ou chimio-embolisation intra-hépatique (CEAH) lorsque le patient a été inscrit sur la liste de TH. Enfin, pour les patients atteints de CHC à un stade précoce et ne pouvant être ni opérés ni transplantés, l'ablation locale par radio-destruction est le traitement standard avec des taux de survie à 5 ans compris entre 50 et 70 % [24, 72]. Dans la vraie vie, la radio-destruction percutanée est devenue la technique principale du traitement curatif du CHC car les résultats sont comparables à ceux obtenus avec la chirurgie de résection, la technique est moins invasive (2 vs 8 jours d'hospitalisation dans notre expérience), et surtout les patients ne doivent pas être nécessairement Child-Pugh A. On peut effectuer une thermoablation par radiofréquence unipolaire ou multipolaire, par micro-ondes, cryothérapie, alcoolisation ou électroporation. Ces techniques sont choisies par les radiologues en RCP, en fonction de la taille de la tumeur et de sa localisation [15, 17]. Ces méthodes sont principalement réservées à des tumeurs de ≤ 3 cm.

### Traitements palliatifs

Les patients atteints de CHC de stade intermédiaire (BCLC B) sont caractérisés par une maladie multinodulaire avec préservation de la fonction hépatique et absence de symptômes liés à la tumeur, d'invasion vasculaire et de maladie extrahépatique. La chimio-embolisation transartérielle (CEAH ou TACE) est recommandée [10, 45]. C'est une technique qui combine la délivrance locale de microbilles (émulsion de lipiodol avec une chimiothérapie, doxorubicine ou cis-platine principalement) embolisant la vascularisation de la tumeur avec l'administration locale de chimiothérapie. La médiane de survie actuelle est de 26 à 40 mois [40, 61].

La radio-embolisation (radiothérapie interne sélective ou SIRT) consiste en l'injection intraartérielle hépatique de microsphères d'yttrium-90 sans effet réel d'embolisation. Elle a été comparée au sorafenib avant que la dosimétrie de la radioactivité délivrée ne soit effectuée [36] : elle n'a pas montré de supériorité mais ses indications sont surtout liées à la possibilité de l'effectuer en cas de thrombose portale et d'adapter la dose radioactive beta à une évaluation initiale *(work up 1)* optimisant l'efficacité de la radiothérapie interne [10, 16, 35].

Chez les patients atteints d'un CHC à un stade avancé (BCLC C), le sorafenib - un inhibiteur de tyrosine kinase - [9] a permis d'augmenter la survie de 7,9 mois à 10,7 mois dans un essai clinique randomisé SHARP [41]. Sur la base de ces données, le sorafenib est devenu le traitement de référence pour ces patients à partir de 2008. Le sorafenib est réservé aux patients non éligibles à un traitement curatif, non répondeurs à deux cures successives de CEAH, OMS 0-2 et Child-Pugh A. Les effets secondaires sont dominés par la diarrhée (39 %), le syndrome palmo-plantaire (21 %) et l'asthénie (22 %) dans l'essai SHARP. L'absence de meilleur traitement peut s'expliquer par la toxicité de certains agents en raison de la cirrhose sous-jacente, l'absence de stratification des patients en fonction de biomarqueurs potentiels et une résistance à la chimiothérapie conventionnelle. Néanmoins, plusieurs médicaments sont actuellement testés dans des essais cliniques de phase III, en association au traitement par sorafenib ou en seconde ligne après progression, tels que des agents anti-angiogéniques (lenvatinib, non remboursé), des inhibiteurs du cycle cellulaire, des inhibiteurs de récepteur de tyrosine kinases. Le régorafénib, inhibiteur de multikinase, s'est avéré être le premier traitement offrant un bénéfice en termes de survie chez les patients atteints de CHC intolérants ou non répondeurs au sorafenib [7]. Aucun inhibiteur de facteur de croissance épidermique (anti-EGFR, erlotinib) [80], ni inhibiteur de phosphatidylinositol 3-kinase / Akt /cible de la voie de la rapamycine (mTOR) (évérolimus) [78], ni inhibiteur de MET (tivantinib) [58], n'a montré de meilleure efficacité que le traitement de référence.

Enfin, les traitements les plus prometteurs aujourd'hui semblent être les immunothérapies qui inhibent les points de contrôle du système immunitaire entre la protéine de mort cellulaire programmée 1 (PD1) et son ligand (PD-L1), et qui montrent un bénéfice en termes de survie chez les patients atteints de nombreux carcinomes métastatiques [48, 71]. Chez les patients atteints de CHC, le taux élevé de PD-L1 sérique était un facteur pronostique péjoratif [27]. Les essais de phase I/II ont montré une utilisation potentiellement bénéfique du nivolumab dans le CHC avancé, avec un taux de réponse objective de 20 % en phase II [23]. Les résultats encourageants du nivolumab n'ont pas été confirmés par l'essai de phase III Checkmate 459 comparant le nivolumab au sorafenib. Au contraire, la supériorité de la combinaison atezo-lizumab + bevacizumab vs sorafenib dans l'essai IMBrave150 a été établie (survie globale et survie sans progression, retard de la dégradation de la qualité de vie) en fait aujourd'hui le traitement de référence [14]. Il est réservé aux patients Child-Pugh A ayant une maladie diffuse hépatique ne relevant pas de traitements palliatifs comme la radio- ou chimio-embolisation ou à ceux ayant une diffusion extra-hépatique.

La radiothérapie externe stéréotaxique et la radiothérapie conformationnelle focalisée à haute dose ont aujourd'hui des indications limitées mais peuvent donner de bons résultats.

Les patients en phase terminale (BCLC D) doivent bénéficier de soins de confort.

L'association française pour l’étude du foie (AFEF) s'est réunie en juin 2023 pour actualiser les recommandations nationales de 2020 sur la prise en charge diagnostique et thérapeutique du carcinome hépatocellulaire [45 et en ligne http://www.tncd.org]. Les protocoles thérapeutiques curatifs ou palliatifs y sont détaillés en fonction des critères liés à la tumeur, à la sévérité de la maladie hépatique sous-jacente et à l’état général du patient. Les progrès, notamment pour les chimiothérapies et leur combinaison éventuelle avec radio-ablation, radio- ou chimio-embolisation, sont tels qu'on ne peut les résumer dans un simple article.

Dans les pays aux ressources limitées pour lesquels nous avons évoqué les limites au dépistage des hépatopathies et du CHC et à leur prise en charge diagnostique (cf. *supra),* les accès aux traitements sont très limités en dehors de la chirurgie de résection dont seule une minorité (< 10 %) peut bénéficier du fait des diagnostics tardifs [29]. Les accès aux autres traitements curatifs (radio-destruction, transplantation) ou palliatifs (radio- ou chimio-embolisation) ne sont souvent pas possibles et pour les chimiothérapies, seul le sorafenib est disponible dans certains pays.

### Traitements spécifiques de l’étiologie de la maladie hépatique

Parallèlement au traitement du CHC, le traitement spécifique de la ou des causes de la maladie chronique du foie sous-jacente doit être associé, afin de réduire le niveau de fibrose et d'améliorer la fonction hépatique. Il est clairement montré que la virosuppression virale B [2, 5, 65, 73], la guérison virale C [11], l'inactivation de l'activité par l'arrêt de l'alcool, le traitement du syndrome métabolique, l'immunosuppression d'une maladie auto-immune ou la chélation martiale des hémo-chromatoses, réduit le risque de progression de la maladie hépatique et de survenue du CHC [11]. Le remodelage de la fibrose et la réversibilité de la cirrhose rendent compte, avec l'amélioration de la fonction hépatique et la réduction de l’HTP et donc des complications non carcinomateuses de la cirrhose, de la diminution progressive du risque de CHC voire de son annulation, selon l'existence ou non de comorbidités hépatiques [44].

En résumé, le fardeau mondial du CHC augmente et il reste de nombreux défis à relever pour améliorer le dépistage et l'accès aux traitements, notamment dans les pays à faibles revenus, et particulièrement en Afrique ou dans certains pays d’Asie.

La prévention reste un élément clé avec la nécessité de développer des actions d'information-éducation-communication sur la prévention des risques de transmission de VHB, VHC et VHD en particulier nosocomiaux et transfusionnels. La vaccination contre le VHB (et par là-même contre le VHD) reste insuffisante alors que son efficacité pour la réduction du risque d'infection et donc d'hépatopathie fibrosante et de cancers est bien établie [68]. Les traitements des infections chroniques à VHB et VHC réduisent significativement l'incidence du CHC [5, 65, 66] mais leur coût reste prohibitif pour les pays à ressources limitées, même si les génériques sont disponibles à faible prix. Des mesures sanitaires sont nécessaires pour réduire l'exposition aux aflatoxines cocarcinogènes. Au-delà de la prévention, l'amélioration du dépistage est cruciale pour permettre une cascade de soins efficace.

Une meilleure compréhension de cette maladie complexe, notamment l'intégration de sousclasses moléculaires de CHC dans le système de stadification clinique, devrait permettre d'offrir le meilleur traitement à chaque patient. Le développement de nouveaux médicaments pour le CHC avancé doit devenir une priorité, avec de nouvelles cibles ou l'amélioration de l'immunité contre les tumeurs : l'identification d'autres biomarqueurs pouvant prédire la réponse aux traitements devrait optimiser l'espérance de vie des patients et réduire le risque de récurrence/progression.

## Financement

Ce travail n'a bénéficié d'aucune source de financement.

## Lien d'intérêt

Orateur ou membre de board pour Gilead, Abbvie, Pfizer, Vivv, LFB.
