# Supplementary material for: Hepatocellular carcinoma (HCC)
Source: Med Trop Sante Int. 2024 Dec 18;4(4):mtsi.v4i4.2024.614. [Article in French] doi: 10.48327/mtsi.v4i4.2024.614 (PMC11892391; doi:10.48327/mtsi.v4i4.2024.614)
Supplement: Related texts

# Research article: Hepatocellular carcinoma (HCC)

## Introduction

Primary liver cancers are tumors that arise from different liver cells. Hepatocellular carcinoma, which develops from hepatocytes, accounts for approximately 75-85% of primary liver cancers [43, 57, 66]. Intrahepatic or extrahepatic cholangiocarcinoma is increasing in incidence and accounts for about 15% of cancers; it develops from bile cells. There are mixed forms of hepatocarcinoma. Hepatoblastoma is a tumor specific to children. Angiosarcoma or epithelioid hemangioendothelioma are rare and originate from the endothelial cell.

Hepatocellular carcinoma (HCC) is the 6^th^ most common cause of cancer worldwide and the 3^rd^ most common cause of cancer-related death [66]. Its incidence is low in northern Europe, but high in sub-Saharan Africa and the Far East, where both hepatotropic viruses and exposure to mycotoxins are high. It complicates cirrhosis in over 90% of cases, with a clear male predominance. The prevalence of HCC is increasing due to improved diagnostic techniques and criteria, an increase in its incidence worldwide [57], as a consequence of alcohol abuse and/or metabolic steatopathies (MASH for metabolic dysfunction associated steatohepatitis) including type 2 diabetes [54, 64], the stability of hepatitis B virus (HBV) infections, which are nevertheless increasing in adults despite vaccination, and hepatitis C virus (HCV) infections. The incidence of HCC is expected to increase over the next 20 years, mainly as a result of MASH, especially in women: MASH has become the leading cause of liver disease worldwide [18, 54]. Infections with HBV or HCV are among the rare human viruses, along with papillomaviruses (HPV), human lymphocytotropic virus (HTLV) and certain herpes family viruses, that are associated with oncogenesis in humans.

The mechanisms of hepatocarcinogenesis remain unclear. Several carcinogenic factors have been identified, but it is difficult to distinguish between initiating and promoting factors. Chronic hepatotropic viral infections, cirrhosis itself, and chemical carcinogens combine to promote the 2-5% annual incidence of hepatocellular carcinoma arising from cirrhosis.

## Epidemiology

Liver cancer is the sixth most common cancer worldwide, the fifth most common in men and the seventh most common in women, accounting for approximately 7% of all cancer diagnoses (approximately 850,000 new cases each year), and its incidence is increasing. It is the third leading cause of cancer death (approximately 800,000 per year) [54, 66]. The annual incidence of liver cancer is very close to the number of deaths per year, underlining the aggressiveness of this disease [66]. Hepatocellular carcinoma (HCC) accounts for 75-85% of all primary liver cancers. The incidence of HCC is higher in men, with a male-to-female ratio of 3 [75]. Worldwide, mortality from HCC is increasing despite the preventable nature of its risk factors.

HCC incidence and mortality are expected to increase in African countries, which are constrained by limited resources to combat endemic levels of viral infection and synergistic environmental risk factors [51]. The changing nature of HCC etiology is particularly illustrated here by traditional risk factors such as viral hepatitis coexisting with a high prevalence of human immunodeficiency virus (HIV) and increasing urbanization, which have fostered a sharp rise in additional risk factors such as co-infection, type 2 diabetes mellitus and obesity [18, 57]. Although there are some differences in etiology between North Africa and Sub-Saharan Africa, risk factors such as chronic viral hepatitis B and C, aflatoxin exposure and iron overload predominate. Aggressive hepatitis B genotypes combined with hepatitis B virus/hepatitis C virus/HIV co-infection and aflatoxin exposure favor a more aggressive molecular phenotype. In addition to a better understanding of the molecular etiology of HCC, policy and planning initiatives to address the burden of HCC must be anchored in the reality of limited available resources. The establishment and coordination of cancer registries across Africa is important to improve the quality of data needed to drive action. Preventive measures, including hepatitis B vaccination programs [68], measures to prevent mother-to-child and childto-child transmission, the provision of universally accessible antiretroviral and antiviral treatment [11, 13], and the reduction of dietary exposure to aflatoxins can make a significant contribution to reducing the incidence of HCC [52]. Finally, the development of biomarkers and new therapeutic interventions will require a better understanding of the unique genetic and epigenetic characteristics of HCC on the African continent.

## Risk Factors

The risk factors for HCC are well established. HCC occurs most frequently in patients with advanced liver fibrosis or cirrhosis, the main risk factor; it is due to chronic liver disease, mainly caused by chronic excessive alcohol consumption, non-alcoholic steatohepatitis MASH, hepatitis B virus (HBV) or hepatitis C virus (HCV) [8]. The highest incidence rates of HCC are found in Asia (China, Mongolia) and sub-Saharan Africa. Overall, 54% of cases could be attributed to HBV infection, 31% to HCV infection and 15% to other causes, but the epidemiology of HCC is evolving: alcohol and MASH remain the most common causes in northern countries and chronic viral infections B, C or D in southern countries [53]. HCC is responsible for the majority of deaths in cirrhotic patients [4].

Cirrhosis results from various mechanisms of liver injury leading to inflammation and fibro-genesis [67]. Histologically, it is characterized by diffuse nodular regeneration surrounded by fibrous septa, with obliteration of the parenchyma leading to distortion of the hepatic vascular architecture. It results in portal hypertension and liver failure [67].

Cirrhosis is the major risk factor for the development of HCC, and approximately 30-35% of cirrhotic patients will develop HCC, with an annual risk of approximately 1-8%, depending on the etiology of cirrhosis [34]. HCC can develop at any stage of cirrhosis, regardless of the cause [24]. The Child-Turcotte-Pugh classification is widely used to characterize the degree of liver damage and the prognosis of patients with cirrhosis [67]. Biannual liver ultrasonography is recommended for early detection of HCC, as treatment of the smallest HCCs is most effective [8, 24, 72]. Metabolic syndrome [47, 72] and tobacco use [37] are co-factors in the risk of liver cancer. Male gender, age > 60 or active disease [24] all contribute to the risk of HCC.

Some protective factors have been described, such as drugs (statins, antidiabetics such as metformin, aspirin) or dietary factors (coffee, vitamin E) [63]. MASH and HBV infection are the two main causes for the development of HCC without cirrhosis [64, 76]. It has been suggested that approximately 40% of patients with HCC on MASH may not have cirrhosis [47].

## Pathophysiology

### Cirrhosis, the major risk factor for HCC

The natural history of HCC in cirrhosis follows a chain of events from the development of precancerous cirrhotic nodules to high-grade dysplastic nodules, which may then lead to the development of early stage HCC (stages 0 and A) and progress to more advanced HCC (stages B and C) [43, 57]. HCC results from the accumulation of somatic oncogene mutations. An accumulation of mutations in the coding regions of approximately 40 somatic functional genes can be observed in HCC nodules, revealing the significant diversity of HCC [59]. Some discriminating markers between precancerous tumor and HCC have been identified: activation of the WNT-ß-catenin pathway, re-expression of fetal genes, deregulation of protein folding mechanisms, response to oxidative stress and maintenance of telomerase [38, 49]. Genetic and epigenetic alterations underlie oncogenic transformation. They can deregulate the expression of oncofetal antigens that become tumor-associated antigens (TAAs) capable of inducing immune responses [28, 55]. Two of these are α-fetoprotein (AFP) and glypican-3 (GPC3). HCC is an inflammation-associated cancer with a very high association (90%) with chronic hepatitis from any cause, including viral infection, excessive alcohol consumption, or MASH, suggesting that the microenvironment plays an important role in the pathogenesis of the disease [30]. Although the presence of an immune infiltrate is associated with a better prognosis [70], activation of nuclear factor kB (NF-kB), epidermal growth factor (EGF) and interleukin-6 (IL-6) in the liver parenchyma and not in tumor is linked to a poor prognosis [31].

Cancer is a DNA disease. The malignant transformation of a cell is secondary to genetic and/ or epigenetic alterations in genes controlling cell cycle, proliferation and survival. The appearance of “advantage” mutations favors the emergence of hepatocyte clones whose increased proliferation favors new mutations.

Mechanisms of hepatocyte transformation can be broadly classified into two groups, depending on whether they emerge in cirrhotic or non-cirrhotic livers. The first group, by far the most frequent, arises in the context of chronic necrotic-inflammatory, genotoxic stress and regeneration. A cell's normal response to DNA damage is cycle arrest. At organ level, the consequence of hepatocyte cycle arrest is a regeneration defect. Sometimes, the natural barriers are crossed, favoring the emergence of more or less dysplastic hepatocyte clones. Genetic alterations observed at this stage include loss or gain of chromosomes leading to alterations in p53 expression, mutations in beta-catenin and AXIN responsible for activation of the Wnt/beta-catenin pathway, and inactivation of pRb1, IGF2R and p16. The development of HCC in cirrhotic patients depends on the activity, duration and etiology of the initial liver disease, and dysplastic nodules and regenerating macro-nodules should be considered as pre-cancerous lesions [8, 75]:

1. the emergence of HCC within cirrhotic nodules is classic (“nodule within a nodule” appearance);
2. 50-60% of cirrhotic macro-nodules have a monoclonal component if the X chromosome methylation profile is studied;
3. chromosomal aberrations and allelic losses are found in half of cirrhotic nodules and in dysplastic nodules.

### HCC without cirrhosis

The rare cases of HCC in healthy livers are specific to certain mechanisms and pathogens: insertional mutagenesis and HBV, R249S mutation of p53 and exposure to aflatoxin B1, kRAS mutation and vinyl chloride, mutation of the transcription factor HNF1a and hepatocytic adenomas, germline mutations of the APC gene and hepatoblastomas. Transformation mechanisms can also be classified according to the presence or absence of chromosomal instability. HBV-associated HCCs are often poorly differentiated and associated with chromosomal instability and frequent mutation of p53. Non-HBV HCCs are often well differentiated, without chromosomal instability, and often associated with mutations in the wnt/beta-catenin pathway.

### Viral hepatocarcinogenesis

#### Epidemiologic Evidence of the HBV/CHC Association

The association between hepatitis B virus (HBV) infection and HCC is suggested by:

1. the increased incidence of this cancer in virally endemic areas, with a relative risk of 10 to 100 in endemic areas [53, 73];
2. animal models of hepadnavirus infection (particularly groundhog virus);
3. the link between initial viral load and HCC [32] and the significant reduction in the incidence of B viral disease due to systematic vaccination policies for newborns and adolescents [5]. Vaccination of newborns of infected mothers, then of adolescents, followed by universal vaccination, has led to an 85% reduction in chronic carriage among newborns of infected mothers and a 50% reduction in the prevalence of chronic HBs antigen carriage in Europe and the United States, as well as in morbidity and mortality related to infection. At the same time, over the last ten years, the incidence of HCC in highly endemic areas (Hong Kong, Singapore, Taiwan) has fallen by around 50% thanks to these vaccination policies [5]. This is the first demonstration of cancer prevention by a vaccine. At the same time, the causal link between the level of viral multiplication and HCC has been clearly established by the Taiwanese “Reveal” study [13] and by the efficacy of virosuppression with nucleoside analogues in reducing the incidence of HCC [2, 65, 68, 73].

#### Mechanisms of viral B hepatocarcinogenesis

The mechanisms of viral B hepatocarcinogenesis are complex [5]. They combine viral genome integration (enabled by a reverse transcription step in HBV replication), chromosomal rearrangements, cis-activation and trans-activation mechanisms. HBV variability has also been implicated (genotype A, deletion in the pre-S2 domain between HBV nucleotides 38-55 [19]).

Cis-activation mechanisms are represented by insertional mutagenesis (cis-activation of a cellular proto-oncogene) described for insertion into the cyclin A gene, the retinoic acid receptor ß gene, and the gene encoding human mevalonate kinase. Trans-activation phenomena are likely: for example, HBV protein X could play a trans-activating role on numerous heterologous or homologous promoters.

Although the mechanisms of viral B hepatocar-cinogenesis are well understood, they are likely to interact with other etiologic factors: alcohol, aflatoxin B1, hepatitis C virus or chronic hepatitis, and cirrhosis itself, as in other hepatopathies, particularly hepatitis C.

#### Viral C hepatocarcinogenesis

Some HCCs have been observed in patients with no detectable HBs antigen (HBsAg), suggesting the involvement of viruses other than HBV, although the identification of integrated HBV sequences in a significant number of cases underscores the possible B viral origin of some HCCs occurring in the absence of HBsAg. Several arguments, mainly epidemiological, suggest that hepatitis C virus (HCV) may promote the emergence of HCC by mechanisms different from those of HBV, due to the absence of integration into the host genome. The greater frequency of antibodies directed against HCV in patients with hepatocellular carcinoma (30-70%) than in the general population (1%), in the absence of detectable HBsAg, is an epidemiological argument. The mechanisms of viral C hepatocellular carcinogenesis remain unknown and are probably multifactorial: the chronic inflammation associated with viral infection and the consequent hepatic fibrosis, the final stage of which is cirrhosis, play an important role. The importance of these factors is illustrated by the fact that in approximately 90% of cases, HCC develops in lesions of chronic hepatitis and cirrhosis.

However, a direct role for HCV is suggested by the in vivo detection of HCV RNA sequences (positive and negative strands) in tumor cells or by the development of HCC associated with capsid expression in the liver of transgenic mice. In vitro, the stable expression of the NS3 non-structural protein transforms NIH 3T3 cells, and expression of the HCV capsid associated with the Ha-ras oncogene could transform rat embryonic fibroblasts. Several HCV viral proteins, including the viral core protein, NS3 and NS5A, have been directly implicated in the transformation and development of HCC [69].

The cellular stress associated with chronic C virus infection activates cellular signaling pathways, such as MAPKs, that facilitate cell proliferation and promote transformation; it leads to the intracellular and extracellular accumulation of mutagenic elements that can induce host cell DNA damage.

#### Viral hepatocarcinogenesis D

Hepatitis D virus (HDV) is a small, defective RNA virus that depends on hepatitis B virus (HBV) for virion assembly and transmission. It replicates in the nucleus of hepatocytes and interacts with several cellular proteins [12]. Chronic hepatitis D is a serious and progressive disease, leading to cirrhosis in 80% of cases and mortality from hepatic decompensation or hepatocellular carcinoma [1, 26]. Whether HDV is an oncogenic virus remains controversial. Studies conducted over the past decade have shown that HDV is associated with a significantly higher risk of developing HCC than HBV monoinfection [1, 12, 26, 62]. However, the mechanisms by which HDV promotes liver cancer, other than the higher prevalence of cirrhosis, remain unclear. Recent data show that the molecular profile of HCC associated with HDV infection is unique and distinct from that of HCC associated with HBV infection, with enrichment of genes involved in cell cycle and DNA replication, and DNA damage and repair, suggesting that genome instability is an important mechanism of hepatocarcinogenesis in HDV [25]. These data suggest that HBV and HDV promote carcinogenesis by different molecular mechanisms, despite the obligatory dependence of HDV on HBV.

### Other carcinogens

Some carcinogens promote liver disease, i.e. fibrogenesis and the risk of HCC (or cholangiocar-cinoma or hepato-cholangiocarcinoma). Alcohol is the major contributor [60]: alcoholic cirrhosis is associated with a 10% risk of developing primary liver cancer. The relative risk among heavy drinkers ranges from 1.2 to 4.2. As a co-carcinogen, excessive alcohol consumption increases the risk of hepatocellular carcinoma due to malnutrition and frequent exposure to hepatotropic viruses associated with chronic alcoholism.

MASH through necrotic-inflammatory activity and fibrosis is another, particularly in the presence of type 2 diabetes [64, 72].

The transcription factor ChREBP appears to be a hepatic oncogene. It is a central metabolic coordinator and, in particular, a key regulator in the transcriptional control of glucose flux through de novo lipogenesis in the liver and white adipose tissue. Deregulation of the functions of this key modulator of inter-organ communication [56] is associated with the development of metabolic diseases such as type 2 diabetes and MASH [3]. Its overexpression in both mouse and human models is involved in the initiation and development of HCC, and its knockdown reduces tumor growth. It may be a new molecular biomarker capable of predicting resistance to systemic treatment with multikinase inhibitors.

Chemical carcinogens have been identified as promoting the development of primary liver tumors. The mycotoxin aflatoxin B1 is the most commonly described; areas of high aflatoxin exposure overlap with areas of high HBV endemicity. There is a probable interaction between chemical carcinogens and hepatotropic viruses. For example, in HBsAg-positive individuals exposed to aflatoxin, aflatoxin B1 has been shown to induce a point mutation in a tumor suppressor or “anti-oncogene” gene: P53 [5, 74].

Other chemical carcinogens such as nitrosamines (in animal models), vinyl chloride (angiosarcoma), or peroxisome proliferators (in animal models) may promote the development of primary liver tumors.

In conclusion, all etiologies of chronic liver disease promote extensive fibrosis and cirrhosis, the major cause of HCC, and HBV (and probably HCV) infection is directly involved in liver carcinogenesis.

In autoimmune hepatitis, which can also progress to cirrhosis, the risk of HCC in this population has recently been shown to be low [20].

Several mechanisms, which are not exclusive, are probably interrelated in the same patient: cirrhosis secondary to chronic active hepatitis or chronic alcohol intoxication is a major risk factor, associated with the tumor in more than 90% of cases. Hepatitis B virus can exert a direct effect by integration with chromosomal rearrangement or insertional mutagenesis, or by transactivation of viral proteins generated from nuclear replicating hepatitis B virus DNA or integrated into cellular DNA. HDV, a satellite of HBV infection, increases the risk of cirrhosis by a factor of 2 to 3 and thus the risk of HCC. HCV can also act directly via viral transforming proteins.

Malignant transformation of hepatocytes generally occurs in the context of chronic liver lysis and regeneration, most commonly in cirrhotic livers. Cell proliferation, inflammation, and genotoxic stress facilitate the accumulation of genetic and epigenetic abnormalities that can:

1. “turn on” oncogenes or “turn off” anti-oncogenes;
2. alter the expression of cell cycle regulatory proteins; 3
3. reactivate telomerase in senescent hepatocytes.

## Diagnosis

### Morphologic diagnosis

HCC is best diagnosed by biannual ultrasonography in patients at risk for extensive fibrosis or previously diagnosed cirrhosis [24, 67, 72]. This early ultrasound detection of HCC, unanimously recommended by professional societies, offers the hope of curative treatment in over 70% of cases. In the absence of known liver disease, the diagnosis may be made by chance or when the patient's general condition deteriorates, revealing both fibrosing liver disease (dysmorphia, even portal hypertension (PH) and/or ascites) and cancer (hypoechoic nodules of specific size and number, as well as permeability of vascular axes, especially the portal trunk). In these cases, the prognosis is worse, and the curability of the tumor is uncertain (37% improvement in 4-year survival in screened patients compared to those not screened [77, 78]).

HCC can be diagnosed by non-invasive (radiology) and/or invasive (biopsy) methods. Radiologic diagnosis is obtained with a high degree of confidence when the lesion occurs in a patient with cirrhosis. Ultrasound leads to dynamic contrast imaging (using dynamic imaging, ultrasound with contrast injection, computed tomography (CT) or magnetic resonance imaging (MRI)): the lesion must meet the radiological criteria for HCC, i.e. hypervascularity in the arterial phase (wash-in) and a decrease in signal relative to the rest of the liver parenchyma in the late phase (wash-out) [46]. When these typical features are present, diagnostic confirmation by biopsy is generally not required [8, 72], especially when alpha-fetoprotein (AFP) is > 250 U/mL. However, advances in our understanding of the molecular biology of tumors may make it essential for prognostic classification and targeted therapies in the future [50]. Biopsy is essential in patients who are not at particular risk of HCC, i.e. patients without cirrhosis in both tumoral and non-tumoral livers.

The recommended algorithm for the evaluation of liver lesions in patients at risk is as follows: for nodules > 1 cm, ultrasound follow-up at 3 months; for lesions > 1 cm, radiological signs of HCC define the diagnosis; if the radiology is atypical on at least one of the two imaging modalities (CT and MRI), radiologically guided biopsy of the tumor and non-tumor liver (mostly echo-guided for echogenic lesions) is recommended [24, 72] (Fig. 1). The difficulties associated with the diagnosis and management of HCC in resource-limited countries must be taken into account. This is all the more unfortunate as the prevalence of HCC is at least 10 times higher than in northern countries for epidemiological reasons (especially HBV) [51]. An evolution in the epidemiology of HCC has been observed in Africa. An analysis of 863 Ivorian patients with HCC diagnosed between 2007 and 2014 showed changes in presentation compared to the period 1970-1980 [22]. The age of presentation is delayed (49.4±14.1 years) and the sex ratio has decreased (M:F=2.6). Hepatitis B surface antigen-positive and anti-HCV-positive patients accounted for 65% and 25% of cases, respectively, while alcohol consumption was reported in 36% of cases. AFP levels were greater than 400 ng/mL in 36% of cases, and tumors were multinodular and/or metastatic at diagnosis in 77% and 26% of patients, respectively. Geographical and anthropological differences were observed, with an excess of female cases in certain regions (Lagunes) or linguistic groups (Kru). Nord Mandé speakers were more often identified as nonB-nonC than others. The epidemiological evolution of HCC in Côte d’Ivoire in recent decades is probably due to the spread of HCV (older, more feminized patient population), which may limit the expected benefits of hepatitis B vaccination and would justify appropriate measures to prevent new HCV transmissions. Delays in diagnosis and treatment explain the considerable differences in the prognosis of HCC between southern and northern countries. The lack of widespread screening for viral liver infections explains the underdiagnosis of chronic viral infections, liver disease and cancer in these settings. There is limited access to serological and genomic diagnostic tests, and few “reflex tests”, i.e. a positive serology - AgHBs, anti-HDV or HCV - leads to a search for anti-HDV and quantification of HBV DNA, HDV or HCV RNA. Assessment of liver fibrosis by non-invasive fibrosis tests (Fibrotest, Fibroscan) is rarely available, although simple, inexpensive tests such as FIB4 should allow assessment of significant fibrosis in the general population and thus identify the risk of cirrhosis and HCC. Finally, access to simple, effective imaging modalities such as ultrasound is not always available. This explains why diagnosis is often made at an advanced stage [22], beyond the possibility of curative treatment. The cost of virological tests or simple biology (alpha-fetoprotein) and the cost of imaging, which is of variable quality, significantly limit the optimization of the care cascade from screening to treatment.

**Figure 1:** Diagnostic approach to hepatocellular carcinoma in the event of the appearance of a liver nodule at the biannual screening ultrasound scan

### Staging

The main prognostic factors for survival in patients with HCC are liver function, tumor characteristics (size and number of HCC nodules, vascular invasion), serum AFP level and general WHO status [24, 72]. The most widely used and accepted staging system in oncology is the staging of malignant tumors (TNM). The problem with this system is that it does not take into account liver function reserve, which is essential in HCC. The European Association for the Study of the Liver (EASL) and the American Association for the Study of Liver Diseases (AASLD) [24, 72] support the Barcelona Classification (BCLC) and recommend the use of this staging system to assess patient prognosis and assign appropriate treatment [39]. The BCLC classification divides patients into five stages (0, A, B, C, D) (Fig. 2) according to predetermined prognostic variables related to

1. the tumor: size and number of HCC nodules, vascular or lymph node invasion, extrahepatic extension as assessed by imaging (chest CT, MRI, more than FDG18 or choline PET scans whose diagnostic acuity is >50%);
2. the patient: general condition, WHO grade and comorbidities;
3. the severity of the biological liver disease (PT, albumin, bilirubin to measure liver function, creatinine edema and Child-Pugh or Meld stage if liver transplantation is contemplated), with or without portal hypertension (PH): platelets, fibroscan or even esogastric endoscopy to look for esophageal or cardio-tuberosal varices according to Baveno criteria.

**Figure 2:** Barcelone staging system (BCLC) and treatment strategy. The classification comprises five stages to select the best candidates for the best therapies currently available. Patients with asymptomatic early tumours (stages 0 - A) are candidates for radical treatment (resection, liver transplantation or local ablation). Asymptomatic patients with multinodular HCC (stage B) require treatment with transarterial chemoembolisation (TACE), while patients with advanced symptomatic and invasive tumours (stage C) are candidates for immunotherapy more than sorafenib. For terminal patients (stage D), only palliative care is offered.

Assessment of the severity of the HCC (hepatic only or more extensive, both hepatic and extrahepatic), the patient's general condition and the severity of his or her liver disease will lead to a treatment decision in a multidisciplinary consultation meeting (MCM).

## HCC Treatments

Treatments are constantly evolving and are collectively decided at the MCM. Depending on the severity of the patient's disease, curative or palliative treatments may be available (Fig. 3).

**Figure 3:** Treatment options according to the BCLC stage of hepatocellular carcinoma

### Curative treatments

These include transcutaneous radioactive destruction of the tumor, resection surgery or liver transplantation. In early-stage disease (BCLC 0 and A), surgical resection is the first-line option for solitary nodules and confers a 70% survival rate at 5 years [6, 24, 72]. Complications (postoperative mortality > 5%) and recurrence have been reduced with restrictive selection of candidates: single nodule, absence of portal hypertension and preserved liver function [24, 54, 72]. HCC recurrence and/or metastases occur in 70% of cases 5 years after resection and currently no adjuvant therapy can prevent this [33].

For BCLC A patients with Milan criteria (single nodule size ≤ 5 cm or up to three nodules up to 3 cm in size, without vascular invasion) and AFP score (size, number of nodules, AFP value) > 2 [21] who are ineligible for surgical resection (liver disease too severe), liver transplantation (HT) is the best first-line treatment [42], with 5- and 10-year survival rates of 70% and over 50%, respectively, and a recurrence rate of about 10% [17, 24, 42, 45, 72]. In reality, 10% of HCCs are eligible for transplantation and less than 5% are transplanted [45]. HCC accounts for one third of the 1,300 THs performed in France each year [45]. TH is most performed after a first recurrence of HCC treated with radioablation or intrahepatic chemoembolization (IHEC), if the patient is on the TH list.

Finally, for patients with early-stage HCC who are ineligible for surgery or transplantation, local ablation by radiodestruction is the standard of care, with 5-year survival rates ranging from 50 to 70% [24, 72]. In the real world, percutaneous radiodestruction has become the main technique for curative treatment of HCC because the results are comparable to those obtained with resection surgery, the technique is less invasive (2 vs. 8 days of hospitalization in our experience), and most importantly, patients do not necessarily have to be Child-Pugh A. Thermoablation can be performed by unipolar or multipolar radiofrequency, microwave, cryotherapy, alcoholization, or electroporation. These techniques are selected by radiologists in RCP depending on tumor size and location [15, 17]. These methods are mainly reserved for tumors > 3 cm in size.

### Palliative treatments

Patients with intermediate stage HCC (BCLC B) are characterized by multinodular disease with preserved liver function and absence of tumor-related symptoms, vascular invasion and extrahepatic disease. Transarterial chemoembolization (CEAH or TACE) is recommended [10, 45]. This technique combines local administration of microbeads (lipiodol emulsion with chemotherapy, mainly doxorubicin or cis-platinum) embolizing the tumor vasculature with local administration of chemotherapy. Current median survival is 26 to 40 months [40, 61].

Radioembolization (selective internal radiotherapy or SIRT) involves intra-arterial injection of yttrium-90 microspheres into the liver, with no real embolizing effect. It has been compared with sorafenib before dosimetry of the radioactivity delivered [36]: it did not show superiority, but its indications are mainly related to the possibility of performing it in the case of portal vein thrombosis and to the adaptation of the beta-radioactive dose to an initial assessment (work-up 1) that optimizes the efficacy of internal radiotherapy [10, 16, 35]. In patients with advanced HCC (BCLC C), sorafenib – a tyrosine kinase inhibitor [9] – prolonged survival from 7.9 months to 10.7 months in the randomized SHARP clinical trial [41]. Based on these data, sorafenib became the reference treatment for these patients in 2008. Sorafenib is reserved for patients who are ineligible for curative treatment and have failed two consecutive lines of CEAH, WHO 0-2 and Child-Pugh A. Side effects were dominated by diarrhea (39%), palmoplantar syndrome (21%) and asthenia (22%) in the SHARP trial.

The lack of better treatment may be explained by the toxicity of certain agents due to underlying cirrhosis, the lack of stratification of patients according to potential biomarkers, and resistance to conventional chemotherapy. Nevertheless, several agents are currently being tested in phase III clinical trials in combination with sorafenib or as second-line therapy after disease progression, including anti-angiogenic agents (lenvatinib, not reimbursed), cell cycle inhibitors and tyrosine kinase receptor inhibitors.

The multikinase inhibitor regorafenib has been shown to be the first treatment to provide a survival benefit in HCC patients intolerant or unresponsive to sorafenib [7].

No epidermal growth factor inhibitor (anti-EGFR, erlotinib) [80], phosphatidylinositol 3-kinase/Akt/rapamycin pathway (mTOR) inhibitor (everolimus) [78] or MET inhibitor (tivantinib) [58] has shown better efficacy than the reference treatment. Finally, the most promising treatments today appear to be immunotherapies that inhibit the immune system's checkpoints between programmed cell death protein 1 (PD-1) and its ligand (PD-L1), which show a survival benefit in patients with many metastatic cancers [48, 71]. In patients with HCC, elevated serum PD-L1 was a poor prognostic factor [27]. Phase I/II studies have demonstrated the potentially beneficial use of nivolumab in advanced HCC, with an objective response rate of 20% in phase II [23]. The encouraging results of nivolumab were not confirmed in the phase III Checkmate 459 trial comparing nivolumab with sorafenib. On the contrary, the superiority of the combination of atezolizumab + bevacizumab over sorafenib was demonstrated in the IMBrave150 trial (overall and progression-free survival, delayed deterioration of quality of life), making it the reference treatment today [14]. It is reserved for Child-Pugh A patients with diffuse liver disease not amenable to palliative treatments such as radio- or chemo-embolization, or those with extrahepatic spread.

Stereotactic external beam radiotherapy and high-dose focused conformal radiotherapy currently have limited indications, but can provide good results.

Patients with end-stage disease (BCLC D) should benefit from comfort care.

The French Association for the Study of the Liver (AFEF) met in June 2023 to update the 2020 national recommendations for the diagnostic and therapeutic management of HCC [45 and online http://www.tncd.org]. Curative and palliative treatment protocols are detailed, based on criteria related to the tumor, the severity of underlying liver disease, and the patient's general condition. Progress, particularly in chemotherapy and its possible combination with radioablation, radio- or chemoembolization, is too great to be summarized in a single article.

In countries with limited resources, for which we have already mentioned the limitations of screening for hepatopathy and HCC and their diagnostic management (see above), access to treatment is very limited, except for resection surgery, from which only a minority (<10%) can benefit due to late diagnosis [29]. Access to other curative (radiodestruction, transplantation) or palliative (radio- or chemoembolization) treatments is often not possible, and for chemotherapy, only sorafenib is available in some countries.

### Treatments Specific to the Cause of the Liver Disease

Along with treatment of HCC, specific treatment of the cause(s) of the underlying chronic liver disease must be combined to reduce the degree of fibrosis and improve liver function. B-virus virosuppression [2, 5, 65, 73], C-virus cure [11], inactivation of activity by alcohol cessation, treatment of metabolic syndrome, immunosuppression for autoimmune disease, or martial chelation for hemochromatosis, have clearly been shown to reduce the risk of liver disease progression and HCC occurrence [11]. The remodeling of fibrosis and the reversibility of cirrhosis, together with the improvement of liver function and the reduction of PH and thus of the non-carcinomatous complications of cirrhosis, account for the progressive reduction or even elimination of the risk of HCC, depending on the presence or absence of hepatic comorbidities [44].

In conclusion, the global burden of HCC is increasing, and many challenges remain to improve screening and access to treatment, particularly in low-income countries, especially in Africa and some Asian countries.

Prevention remains a key element, with the need to develop information-education-communication initiatives to prevent the risks of HBV, HCV and HDV transmission, particularly nosocomial and transfusion-related. Vaccination against HBV (and consequently against HDV) remains inadequate, although its efficacy in reducing the risk of infection and thus of fibrosing liver disease and cancer is well established [68]. Treatments for chronic HBV and HCV infection significantly reduce the incidence of HCC [5, 65, 66], but their cost remains prohibitive for resource-limited countries, even when generic versions are available at low prices. Public health interventions are needed to reduce exposure to cocarcinogenic aflatoxins. In addition to prevention, improved screening is essential to enable an effective cascade of care.

A better understanding of this complex disease, including the integration of molecular subclasses of HCC into the clinical staging system, should enable the best treatment to be offered to each patient. The development of new drugs for advanced HCC must become a priority, with new targets or improved tumor immunity: the identification of other biomarkers that can predict response to treatment should optimize patient life expectancy and reduce the risk of recurrence/progression.

## Funding

No funding was provided for this work.

## Links of interest

Speaker or board member for Gilead, Abbvie, Pfizer, Vivv, LFB.
